# Increased CCL-5 (RANTES) Gene Expression in the Choroid Plexus of Dogs with Canine Leishmaniosis

**DOI:** 10.3390/ani13132060

**Published:** 2023-06-22

**Authors:** José Eduardo dos Santos Silva, Giulia Gonçalves Jussiani, Fernanda Grecco Grano, Maria Cecília Clarindo Pelissari, Guilherme Dias de Melo, Tatiane Terumi Negrão Watanabe, Valéria Felix de Lima, Gisele Fabrino Machado

**Affiliations:** 1 School of Veterinary Medicine, Faculdade de Medicina Veterinária de Araçatuba (FMVA), São Paulo State University (UNESP), R. Clóvis Pestana, 793, Dona Amélia, Araçatuba 16050-680, SP, Brazil; 2 Lyssavirus Epidemiology and Neuropathology Unit, Intitut Pasteur, Université Paris Cité, 75015 Paris, France; 3 Department Population Health and Pathobiology, College of Veterinary Medicine, North Carolina State University, Raleigh, NC 27607, USA; 4 Antech Diagnostics, 12401 West Olympic Blvd, Los Angeles, CA 90064, USA

**Keywords:** cerebrospinal fluid, central nervous system, neuroinflammation, chemokines

## Abstract

**Simple Summary:**

The choroid plexus is a specialized structure responsible for the production and secretion of cerebrospinal fluid, and it is considered an interface between the peripheral immune system and the central nervous system. It can allow the passage of inflammatory cells or pathogens and has the potential to act as a source of inflammatory mediators in several diseases. Thus, this study aimed to evaluate the role of the choroid plexus as a possible route of inflammatory cells in the development of brain lesions in dogs with canine leishmaniosis, as well as its association with blood–cerebrospinal barrier dysfunction. Our findings demonstrated blood–cerebrospinal barrier dysfunction during leishmaniosis and suggest that the chemokines CCL-5 and CXCL-10 can be responsible for the recruitment of inflammatory cells found in choroid plexus.

**Abstract:**

Visceral canine leishmaniasis (CanL) can cause several clinical manifestations, including neurological lesions. Few reports have characterized the lesions observed in the central nervous system (CNS) during CanL; however, its pathogenesis remains unclear. The choroid plexus (CP) is a specialized structure responsible for the production and secretion of cerebrospinal fluid (CSF) and considered an interface between the peripheral immune system and CNS. It can allow the passage of inflammatory cells or pathogens and has the potential to act as a source of inflammatory mediators in several diseases. Thus, this study aimed to evaluate the role of CP as a possible route of inflammatory cells in the development of brain lesions in dogs with CanL, as well as its association with blood–CSF barrier (BCSFB) dysfunction. Samples were collected from 19 dogs that were naturally infected with CanL. We evaluated the histopathological lesions in the brain and investigated the gene expression of the cytokines. Capture enzyme-linked immunosorbent assay (ELISA) was used to evaluate the presence of the same cytokines in the CSF. Biochemical analysis was performed to compare the presence of albumin in the serum and CSF. Indirect ELISA was performed to measure the presence of anti-Leishmania antibodies in the CSF, which would suggest the disruption of the BCSFB. Histopathological evaluation of the dogs’ brains revealed mild-to-severe inflammatory infiltrates, mainly in the CP and meninges. We also detected the presence of anti-Leishmania antibodies and albumin in the CSF, as well as Leishmania DNA in the CP. The gene expression of CCL-5 was increased in the CP of infected dogs compared with that of controls, and there was a tendency for the increase in the gene expression of CXCL-10. Thus, our findings confirm the disfunction of the BCSFB during CanL and suggest that the chemokines CCL-5 and CXCL-10 can be responsible for the recruitment of inflammatory cells found in CP.

## 1. Introduction

Leishmaniasis is a zoonotic, chronic, and systemic disease caused by several protozoan *Leishmania* spp. It is endemic in 76 countries and has been described in at least 12 countries on the American continent. Brazil registered 90% of the cases in Latin America, reporting 1.993 human cases in 2020, with 165 deaths (Ministry of Health, Brazil, 2022). In cities, dogs play a key role as domestic reservoirs for parasites [1].

Visceral canine leishmaniasis (CanL) can cause several clinical manifestations in dogs, of which the most common are generalized lymphadenopathy, cachexia, lethargy, hepatosplenomegaly, fever, and chronic diarrhea [2]. Neurological alterations, such as seizures, motor incoordination, paresis, and myoclonus, have already been reported [3,4,5,6]. However, studies have demonstrated that only 4–5% of chronically infected dogs develop neurological signs [6,7], although histopathological alterations in the central nervous system (CNS) are frequently described [8].

Naturally infected dogs have demonstrated evidence of brain inflammation and disruption of the blood–brain barrier (BBB) [8,9], presenting with alterations such as meningitis, choroiditis, perivascular immunoglobulin deposition, and signs of glial activation [10]. The activation of enzymes that degrade extracellular matrix components and pro-inflammatory profile of cytokines and chemokines in the brain have also been detected [11,12,13]. 

The choroid plexus (CP) is a specialized structure responsible for the production and secretion of the cerebrospinal fluid (CSF). It is composed of a single layer of highly vascularized epithelial cells located in the lateral, third, and fourth ventricles. These cells have occlusive-type intercellular junctions that form the blood–cerebrospinal barrier (BCSFB). Since the BBB is absent in the CP, the BCSFB is responsible for avoiding direct contact between the blood and CSF; nevertheless, access of blood molecules and cells into the choroidal stroma occurs. Thus, the CP is considered to be a mediator between the peripheral immune system and CNS, allowing the passage of inflammatory cells and acting as a producer of inflammatory mediators in several diseases [14,15,16]. In addition, the CP and CSF can act as entry sources for circulating pathogens and antigens [17]. 

It is already know that epithelial cells from the CP have the ability to produce cytokines, such as interleukin (IL)-6, IL-8, tumor necrosis factor α (TNF-α), and IL-1β, which can be released into the CSF when systemic peripheral inflammation occurs, resulting in changes in the nervous tissue [16,18,19,20]. Regarding chemokines, few studies have been conducted on their synthesis and secretion by CP [21]. However, CP epithelial cells are believed to express chemokines and adhesion molecules that facilitate the homing of leukocytes from the CSF to the CP [22,23]. 

Macrophage inflammatory protein-1α (CCL-3) and Motif chemokine ligand 4 (CCL-4) are chemokines expressed by activated inflammatory macrophages, whereas Motif chemokine ligand 5 (CCL-5), also known by “Regulated on Activation, Normal T cell Expressed and Secreted” (RANTES), and C-X-C motif chemokine ligand 10 (CXCL-10) are expressed and secreted by T lymphocytes [24]. CCL-5 and CXCL-10 are associated with regulating the migration of Th1 effector cells to the site of inflammation. Th1 response in leishmaniasis is associated with parasite control and elimination [25,26].

Previous studies conducted by our research group pointed out a pro-inflammatory brain environment favorable to an accumulation of T lymphocytes CD3+ in dogs infected with CanL, especially in leptomeninges, the subependymal region, and the CP [8,9]. Histopathological evaluation of the CP also demonstrated changes that suggested dysfunction in the BCSFB [9]. From these findings, in this study, we evaluated the CP with the aim of determining its possible role as a gateway to the parasite and as structure responsible for producing pro-inflammatory mediators, triggering and perpetuating the inflammatory process within the nervous environment. Therefore, we evaluated the expression of cytokines and chemokines already known to be differentially expressed in the brains of dogs with CanL [11,27,28] and assessed BCSFB disfunction by measuring the presence of albumin and anti-*Leishmania* antibodies in the CSF. 

## 2. Materials and Methods

### 2.1. Sample Collection

A total of 19 adult dogs, non-bred; aged between one and five years; in a random proportion of males and females; not vaccinated against leishmaniasis; serologically positive for CanL as shown by enzyme-linked immunosorbent assay (ELISA; cut-off > 0.270); and presenting characteristic symptoms of the disease, such as weight loss, lymphadenopathy, dermatological lesions and/or onychogryphosis, were included in the infected group. 

The control group consisted of four dogs, negative for CanL as shown by ELISA and quantitative polymerase chain reaction (qPCR), and whose cause of euthanasia was related to complications of trauma lesions, showing no signs related to infectious or neurological diseases.

The dogs of both groups were euthanized according to the protocol of the Federal Council of Veterinary Medicine of Brazil, in the Center for Zoonosis Control of Araçatuba. Before euthanasia, they were anesthetized with sodium pentobarbital (Hypnol^®^ 3%) at a dose of 15–20 mg/kg, intravenously (IV). Under anesthesia, blood and CSF samples were collected for biochemical testing, allowing clinical staging of the animals according to Solano-Gallego et al. (2009) [2]. Subsequently, potassium chloride (19.1%) was administered at a dose of 2 mL/kg, IV. All of the dogs were serologically negative for *Neospora caninum* and *Toxoplasma gondii*, as shown by the indirect immunofluorescence reaction (IFA, tilters up to 1:16 were considered negative), and were negative in blood PCR for *Babesia* spp. and *Ehrlichia* spp. 

Routine necropsy was performed and samples from the spleen and brain were collected and immediately stored frozen in RNA preserving solution (RNAlater, Applied Biosystems, AM7020) or 10% formaldehyde, according to the needs of the techniques to be performed. 

For histopathological evaluation of the brain, samples of coronal sections of the cortical regions (temporal and piriformis), hippocampus, and thalamus, containing the periventricular region, were obtained, including the CP. 

**Figure 1:**
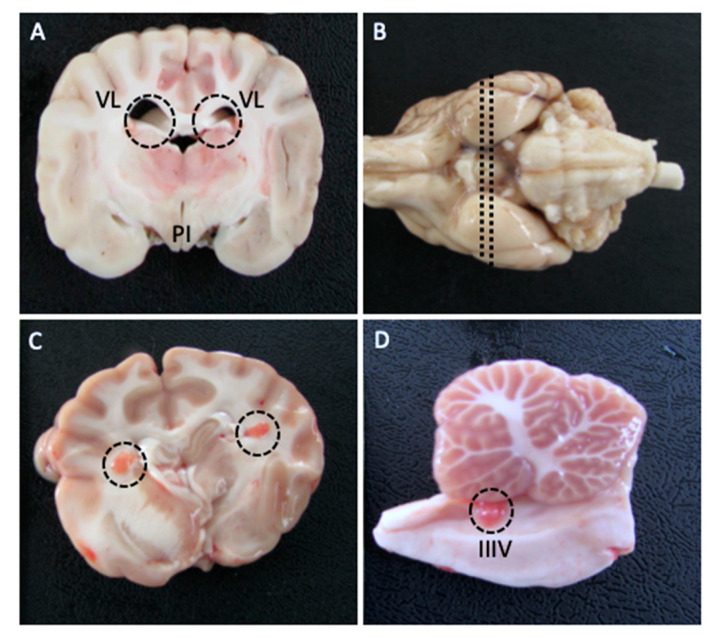
Coronal section highlighting the lateral ventricles (VL) (dashed circles) and pituitary infundibulum (PI) (**A**). In the lateral ventricles, the choroid plexus can be visualized as a red structure in non-fixed tissue ((**C**)—dashed circles – Figure Caption). (**B**) Median sagittal section of the cerebellum and brainstem, at the level of the cerebellar peduncles, passing through the infundibulum. The choroid plexus of the fourth ventricle (IIIV) is highlighted in the dashed circle (**D**).

For molecular analysis of the CP, in order to collect the structure in the lateral and fourth ventricles, a coronal section passing through the center of the pituitary infundibulum and a cranial coronal section to the infundibulum, passing through the mamillary bodies, were made (Figure 1A). The choroid plexus of the fourth ventricle was identified after performing a median sagittal section of the cerebellum and brainstem, at the level of the cerebellar peduncles (Figure 1B). In non-fixed tissue, the red coloration of the structure was easily visualized, allowing us to efficiently isolate and collect fragments of the CP (Figure 1C,D). 

### 2.2. Histopathological Evaluation of the Brain

The intensity of brain inflammation in hematoxylin and eosin-stained tissues was determined semiquantitatively using a 0–3 scoring system, as previously described by Melo and Machado [8]. The presence and intensity of the inflammatory infiltrate was assessed in the leptomeninges, subependymal region, and CP. The absence of inflammatory cells was assigned a score of 0; discreet or focal accumulation of rare cells was assigned a score of 1; diffuse or multifocal accumulation of few cells was assigned a score of 2; and the marked presence of inflammatory cells was assigned a score of 3. The presence of perivascular cuffs was also evaluated, and only cuffs with at least two layers of inflammatory cells around the vessel were considered.

### 2.3. Detection and Quantification of Leishmania DNA in the Spleen and CP

Total DNA was extracted from tissue fragments of the spleen and CP using the DNeasy Blood & Tissue Kit (69506, Qiagen, Hilden, Germany) according to the manufacturer’s protocol. Spleen qPCR was performed to confirm the positivity of infected animals as well as to check their parasite load. Quantification of Leishmania DNA in CP was performed only in 13 dogs in the infected group due to the small amount of material. The extracted DNA was quantified using a NanoDrop spectrophotometer (260/280 ratio of 1.8 and 2.0). qPCR was performed using Eppendorf Mastercycler^®^ RealPlex2, SYBR Green PCR Master Mix (4309155, Applied Biosystems) and 900 nM of each primer (sense: 5′-CCTATTTTACACCAACCCCCAGT-3′; anti-sense: 5′-GGGTAGGGGCGTTCTGCGAAA-3′), which amplify a 116 bp fragment of the minicircle kinetoplast DNA (kDNA) of *Leishmania* spp. [29]. Duplicate samples were incubated at 94 °C for 2 min, followed by 40 amplification cycles (94 °C for 15 s, followed by 60 °C for 1 min). Subsequently, the samples were subjected to a dissociation curve (melt curve) from 60 to 95 °C, with increments of 0.5 °C every 5 s. Nuclease-free water was used as the negative control for the reaction. For absolute quantification, serial dilutions from 10^−1^ to 10^6^ L. infantum DNA (MHOM/BR/72/LD46) were used to construct a standard curve. 

### 2.4. Cytokines and Chemokines Gene Expression in the CP

CP samples were extracted using the RNeasy^®^ Lipid Tissue Mini kit (74804, Qiagen, Hilden, Germany) following the manufacturer’s recommendations. After obtaining RNA, genomic DNA lysis was performed using the RNase-Free DNase Set (79254; Qiagen). Total RNA was quantified using a NanoDrop spectrophotometer (260/280 ratio, 2.0 and 2.3). Reverse transcription was performed to obtain cDNA using the RT2 First Strand Kit (330404; Qiagen) following the manufacturer’s instructions. qPCR was performed using the CSFX96TM Real-Time System (Bio-Rad^®^, Hercules, CA, USA) detection system with the commercial Taqman^®^ Universal Mastermix (4326708, Applied Biosystems, Waltham, MA, USA). 

The concentrations of the oligonucleotide primers and probes for IL-1β, IL-6, TNF-α, interferon γ (INF-γ), and glyceraldehyde-3-phosphate dehydrogenase (G3PDH) were determined according to the methods described by Fujiwara et al. [29] and Peters et al. [30]. CCL-3, CCL-5, CXCL10, and ribosomal protein L32 (RPL-32) were purchased from Life Technologies (Carlsbad, CA, USA). The amplification conditions were: 55 °C for 2 min, 95 °C for 10 min, 45 cycles at 95 °C for 15 s, and 60 °C for 1 min. Reactions were performed in duplicate. 

The quantification of cytokines and chemokines gene expression was evaluated using the 2-ΔΔCt method, as previously described by Livak and Schmittgen (2001) [31], using the geometric mean of two reference gene signals, G3PDH and RPL-32, for data normalization, as described by Pfaffl et al. (2004) [32]. The results were described using relative gene expression, which indicates the number of times (fold change) the expression of a target gene was higher (upregulated) or lower (downregulated) in the infected group than in the control group.

### 2.5. Cytokines in the Serum and CSF

Capture ELISA was performed according to manufacturer’s instructions to detect and quantify the presence of cytokines IFN-γ, IL-1β, TNF-α, and IL-6 in the CSF and serum of infected dogs. CSF and serum samples were centrifuged at 10,000× *g* for 15 min at 4 °C, and the supernatant was separated and immediately stored at −80 °C. The concentrations of TNF-α, IFN-γ, and IL-1β were determined using commercial kits (Duo SET^®^ Canine TNF-α; Duo SET^®^ Canine IFN-γ and Duo SET^®^ Canine IL-1β—R&D System, Minneapolis, MI, USA).

For IL-6 evaluation, anti-canine monoclonal antibody (mAb) produced in mice (Cat. Number: MAB16091, R&D Systems, USA) and biotinylated anti-canine polyclonal antibody produced in goat (Cat. Number: BAF1609, R & D System, USA) were used. Plates with 96 wells (Corning, NY, USA) were sensitized with 2 μg/mL of mAb and 1 μg/mL of detection antibody. Recombinant canine IL-6 (Cat. Number: 1609-CL, R&D Systems, USA) was used to generate the standard curves. The test was developed using 3,3′,5,5′-tetramethylbenzidine (TMB; Sigma, Ronkonkoma, NY, USA). 

Samples were measured in duplicate, and the plates were read using a spectrophotometer (Spectra Count, Packard Bio Science Company, Meriden, CT, USA) at 450 nm. The limit of detection was 3.90 pg/mL in CSF and 7.81 pg/mL in serum for IL-1β, 78.12 pg/mL in CSF and 9.7 pg/mL in serum for IL-6, 31.21 pg/mL in CSF and 15.62 pg/mL in serum for TNF-α, and 15.62 pg/mL in CSF and 62.5 pg/mL in serum for IFN-γ.

### 2.6. Albumin and Anti-Leishmania Antibodies in the CSF

Biochemical tests were performed to assess the levels of total protein and albumin in the serum and CSF to verify the possible breakdown of the barriers (BBB and BCSFB). The albumin quotient was calculated using the formula established by Gama et al. (2007) [33] and values higher than 0.64 were considered positive for loss of the blood CSF barrier integrity. Indirect ELISA was used to determine the concentration of anti-Leishmania antibodies in the CSF, as previously described by Lima et al. (2003) [34]. The assays were performed in only 16 dogs because of the restricted amount of CSF. 

### 2.7. Statistical Analysis

Statistical analyses were performed using Prism software (v6.05, GraphPad, La Jolla, CA, USA). Differences between the infected and control groups were determined using the Mann–Whitney test, and correlation was assessed using the Spearman test. Statistical significance was set at *p* < 0.05. Evidence of statistical trend was considered when 0.05 < *p* < 0.1.

## 3. Results

### 3.1. Clinical Staging

As previously described by Solano-Gallego et al. [2], 26.31% (5/19) of the dogs were classified in stage I, 52.63% (10/19) in stage II, 15.78% (3/19) in stage III, and 5.26% (1/19) in stage IV. The animals’ data and test results that allowed staging are available in Supplementary Table S1 in the Supplementary Materials. 

Macroscopic evaluation revealed that 78.94% (15/19) of the infected dogs presented with weight loss, while 68.42% (13/19) had skin alterations. Splenomegaly was observed in 63.15% (12/19) of the dogs, and 35.29% (7/19) had generalized lymphadenopathy. Laboratory analyses showed that 84.21% (16/19) of the infected dogs had anemia, 52.63% (10/19) had lymphopenia, and 31.57% (6/19) had thrombocytopenia. Almost all infected dogs showed hypoalbuminemia. Moreover, azotemia was evident in 31.57% (6/19) of the infected dogs.

### 3.2. Brain Histopathology and Immunohistochemistry

**Figure 2:**
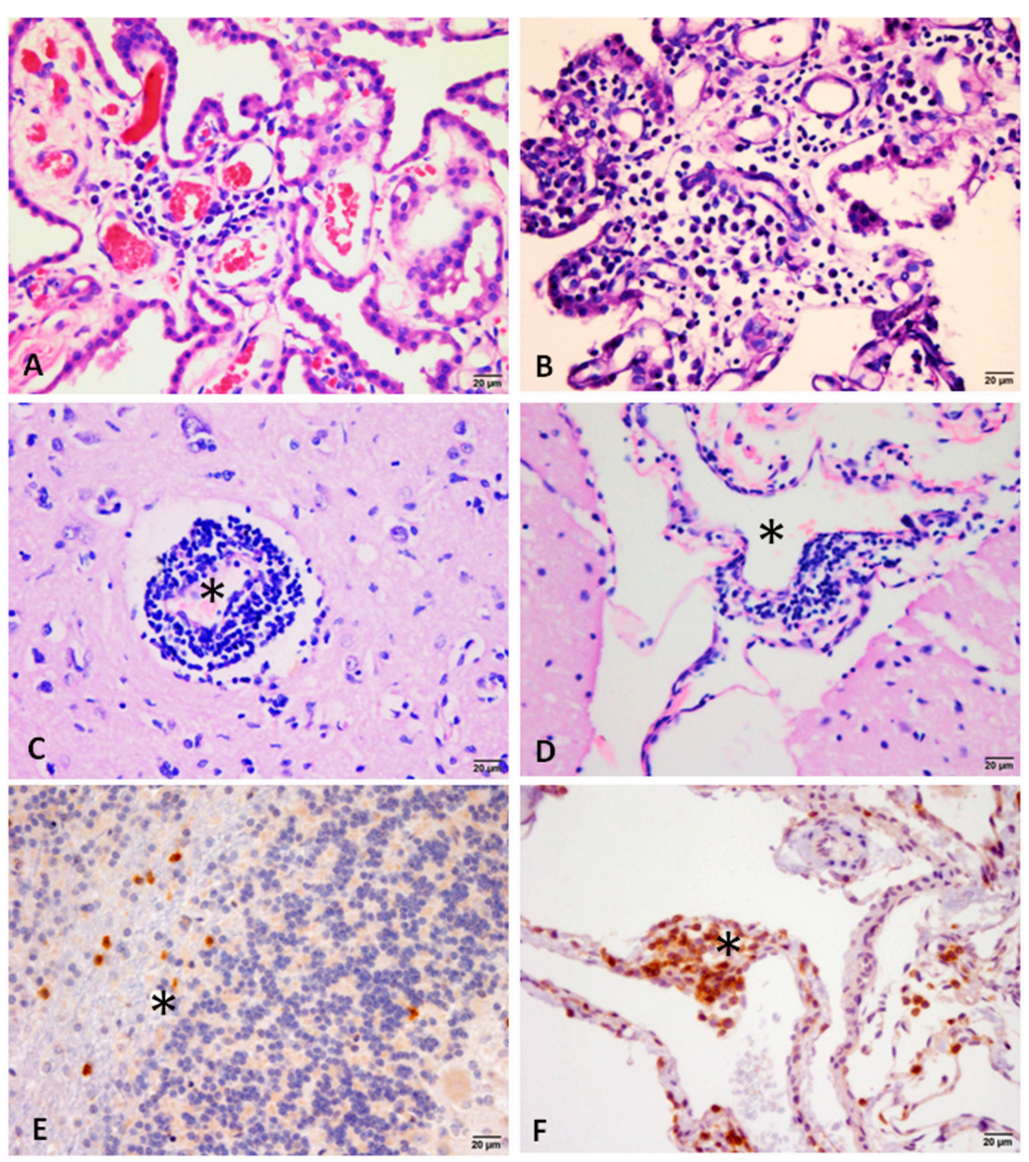
Representative photomicrographs of the inflammatory changes observed in the brain of dog with visceral leishmaniasis. (**A**) Inflammatory infiltrate of mononuclear cells with focal distribution in the CP. (**B**) Plexus choroiditis characterized by marked mononuclear inflammation and evident stromal thickening. (**C**) Perivascular cuff (*) with more than two layers in the region of the diencephalon. (**D**) Focal perivascular lymphoplasmacytic infiltrate (*) in the leptomeninges. (**E**,**F**) Immunolocalization of CD3+ T lymphocytes in the cerebellar white matter (*)composing the inflammatory infiltrate of the leptomeninges (*) ((**A**–**D**) HE stain and (**E**,**F**) immunostain for CD3 lymphocytes, bar = 20 μm).

**Figure 3:**
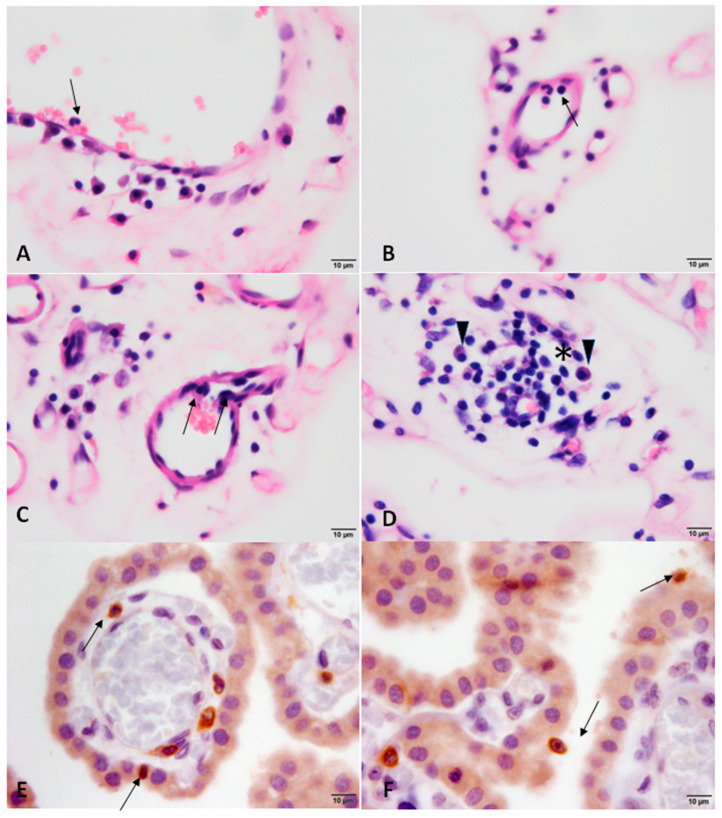
Photomicrographs of choroid plexus (CP) of dog with visceral leishmaniasis (**A**–**C**). Note predominantly mononuclear inflammatory cells (lymphocytes and plasma cells) marginalizing the endothelial cells and transmigrating through the capillaries (arrows). Arrow in (**B**) also points out a rarely observed neutrophil. In (**D**), perivascular infiltrate around a capillary of the VC (*), with lymphocytes and plasma cells (arrowhead). In (**E**,**F**), perivascular CD3+ T lymphocytes, transmigrating through the epithelium and adhered to the apical surface of the PC epithelial cells ((**A**–**D**) HE stain and (**E**,**F**) immunostain for CD3 lymphocytes, bar = 10 μm).

**Figure A1:**
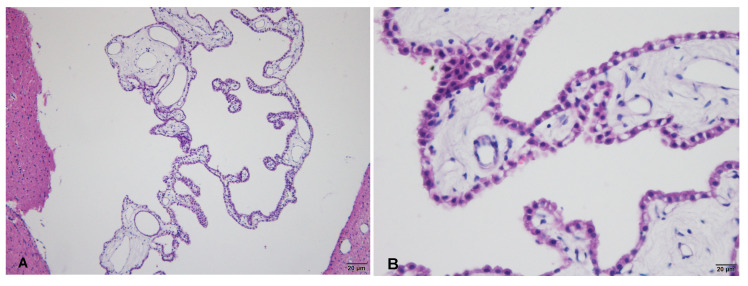
Unaltered choroid plexus from control dogs ((**A**) Obj. 20×; (**B**) Obj. 40×, hematoxylin–eosin staining).

Mild-to-severe inflammation was the most common change found in the brains of the infected dogs, which was frequently in the leptomeninges and CP. Representative images of inflammation in each evaluated area are shown in Figure 2. In addition, perivascular cuffing was present in 26.31% (5/19) (Figure 2C). The inflammatory infiltrate was composed predominantly of lymphocytes and plasma cells (lymphoplasmacytic). It is possible to observe the margination and transmigration of leukocytes in the capillaries of the choroid plexus on HE staining (Figure 3A–D), and the participation of TCD3+ lymphocytes was confirmed by immunohistochemistry (Figure 3E,F). Amastigote forms of *Leishmania* were visualized in the CP of one dog [35]. Table 1 summarizes the percentage of infected animals with brain inflammation and its intensity in each region evaluated. Supplementary Table S2 in the Supplementary Materials details the histopathological findings of each dog in the study. Appendix A, Figure A1, demonstrates the histology of the choroid plexus with minimal changes, belonging to the control dogs.

### 3.3. Detection and Quantification of Leishmania DNA in the CP

The presence of kDNA from *L. infantum chagasi* was detected in the spleens of all dogs and in the CP of 38.46% (5/13) of the dogs with CanL. The amplification reaction obtained an efficiency of 106%, coefficient of determination (r2) of 0.968, and angular coefficient of −3.179. The standard curve of the serial dilutions of *L. infantum* DNA allowed for the quantification of the parasite load in PC, obtaining a detection range of 1 to 27,778 parasites per 10 mg of CP. 

### 3.4. Gene Expression of Cytokines and Chemokines in the CP

**Figure 4:**
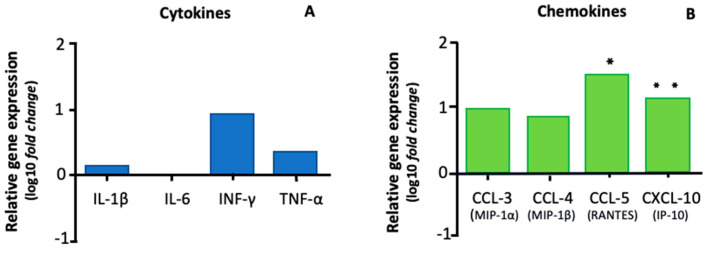
Mean relative gene expression of cytokines (**A**) and chemokines (**B**) in the choroid plexus of dogs with visceral leishmaniasis. The values, on the logarithmic scale at base 10, indicate the number of times (fold change) the gene for cytokines and chemokines is more (positive values) or less expressed (negative values) in relation to the dogs in the control group. The normalization factor was the reference gene, glyceraldehyde 3-phosphate dehydrogenase, and ribosomal protein L32 gene according to the 2^−ΔΔCt^ method. * Indicates *p* < 0.05, ±0.05 < *p* < 0.1; ** indicates tendency for increased gene expression.

The gene expression of the cytokines IL-1β, IL-6, INF-γ, and TNF-α in the CP of dogs with CanL showed no statistical difference compared with the control group (Figure 4A). Meanwhile, the gene expression of the chemokine CCL-5 in the CP of dogs with CanL was higher than that in the control group (*p* < 0.05), whereas that of CXCL-10 tended to increase (*p* = 0.05) (Figure 4B)**.** However, there was no statistical difference in the gene expression of CCL-3 and CCL-4 compared with the control group.

### 3.5. Cytokines in the Serum and CSF

The cytokines IL-6 and IFN-γ were not detected in the serum or CSF of any of the dogs with CanL in this study. IL1-β was detected in the serum and TNF-α in the CSF; however, there was no statistical difference with the control group. 

### 3.6. Albumin and Anti-Leishmania Antibodies in the CSF

**Figure 5:**
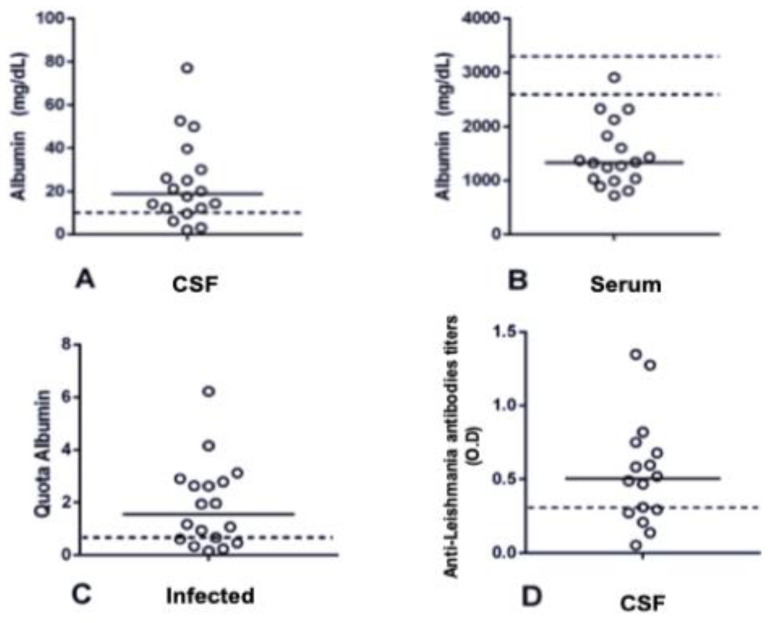
Determination of albumin concentration and quotient in the serum and cerebrospinal fluid (CSF) and anti-Leishmania antibodies in the CSF of dogs with CanL. Horizontal line indicates the median. The dotted lines represent the cut-off values. White circles represents the result of each dog. (**A**) CSF albumin of infected dogs with CanL (reference value 2600–3300 mg/dL). (**B**) Serum albumin of infected dogs with CanL (reference value < 10 mg/dL). (**C**) Albumin quota established in the CSF of infected dogs with CanL (reference value > 0.64). (**D**) Concentration of specific anti-Leishmania antibodies in the CSF of infected dogs with CanL. O.D., optical density.

Anti-Leishmania antibodies were detected in the CSF samples of 68.75% (11/16) of the dogs infected with CanL. The optical density ranged from 0.1 to 1.348 (Figure 5D). An increase in the amount of albumin in CSF was observed in 77.77% (14/18) of the dogs infected with CanL compared with that in healthy dogs (Figure 5A). The albumin quotient was increased in 72.22% (13/18) of the dogs infected with CanL (Figure 5C).

## 4. Discussion

Histopathological analysis of brain samples from dogs with CanL demonstrated the presence of mononuclear lymphoplasmacytic inflammatory cells that were mostly located in perivascular areas, mainly in the leptomeninges of the cortical, cerebral, and cerebellar regions, as well as in the subependymal region and CP, with different intensities. This finding is consistent with that of our previous studies and those of others that characterized histopathological lesions in the brain of dogs with leishmaniasis, which can be present even in the absence of neurological signs [8,9,36]. Similar to other studies [4,9], we did not find a correlation between the intensity of inflammation and the clinical staging of the animals. 

The CNS has a low number of inflammatory cells under normal conditions and it is considered an immunologically privileged region with a complex system of protective barriers [37]. In the CP, macrophages, dendritic cells, and rare lymphocytes are expected to be found in the matrix stroma, responsible for antigen presentation and immunosurveillance in the region [38]. T cell lymphocytes, after peripheral immune stimulation, adhere to the CP and transmigrate through its epithelium, where they can be activated and proliferate, based on the stimulation of specific antigens [23]. We suggest that the inflammatory infiltrate observed mostly in the CP of CanL infected dogs indicates an active process of cell migration and proliferation through the CP. Systemic inflammatory mediators released during CanL infection, as well as the presence of the parasite itself, are possible mechanisms that can trigger immune cell trafficking to the CNS, passing through the CP. The presence of plasma cells in the infiltrate has been described in various organs and tissues in CanL [39].

We detected Leishmania DNA via qPCR in the CP of 38% (5/13) of the infected dogs. The presence of parasite DNA in the CSN and CSF using qPCR has been previously reported (8,35), albeit the presence of amastigotes forms were observed in the CP of only one dog, by immunohistochemistry, and this result was published in a previous paper [35]. To the best of our knowledge, there are only a few reports on the presence of the whole parasite in the CNS of dogs [36], including amastigotes found in the CP [40], meninges [41], and spinal cord [41,42]. The highest CP parasite load was found in the sample from the same dog in whose CP we observed amastigote forms of the parasite via immunohistochemistry. It is likely that all dogs with a positive PCR in CP samples present amastigotes in the CP, undetected by immunohistochemistry, but detected by PCR, a more sensitive test. It is unlikely that the *Leishmania* DNA identified in the CP comes from the blood, considering that not all infected animals, thus blood positive, were also PCR positive in the CP. 

Presence of anti-*Leishmania* antibodies and albumin in the CSF of infected dogs in this study corroborates the hypothesis of a brain barrier dysfunction promoted by *Leishmania* infection, already observed in the BBB [8,9] and now in the BCSFB. In response to systemic inflammation, inflammatory mediators have direct access to the brain through the circumventricular organs, as well as through the disrupted brain barriers, which allow the penetration of various mediators and potential neurotoxic factors into the brain [14,37,43]. 

In our study, no statistical difference in the gene expression of the cytokines IL-1β, IL-6, TNF-α, and IFN-γ was detected in the CP of dogs with CanL compared with control dogs. Furthermore, these cytokines were not detected in the serum and CSF of the dogs, except for IL1-β in the serum and TNF-α in the CSF, and they did not differ from control dogs. 

Studies which evaluated cytokine gene expression in the brains of dogs with CanL showed an increased expression of IL-1β, IL-6, TNF-α, and IFN-γ [11,27]. As we did not observe a similar pattern in the CP of infected dogs, this suggests that the CP does not directly participate in the production of these specific cytokines. Notwithstanding, the CP is constantly exposed to signals from the brain parenchyma and peripheral immune system; therefore, its role in CanL may be restricted to mediating the inflammatory cell transmigration to the CSF and the inflammatory response [14,17,23]. 

The presence of Leishmania DNA in the CP, as well as peripheral inflammatory mediators induced by infection, could activate local Toll-like receptors, such as TLRs 2 and 9, which are increased in the CP of dogs with CanL [28], and then trigger the production of cytokines and chemokines in other CNS cells of infected dogs [11,27], such as microglia and astrocytes, which are absent in the CP, but exhibit an activated phenotype in CanL. Glial cell activation produces cytokines and others inflammatory mediators that can aggravate barrier dysfunction [10]. 

Systemic inflammatory stimuli resulting from *Leishmania* infection could promote a compartmentalized and CNS-specific inflammatory response, as the immune response to leishmaniasis in different organs is distinct, even after the same systemic peripheral stimulus [39,44]. In this case, studies suggest that the CP, after peripheral stimulation, acts by promoting T cell trafficking through the CNS and modulating the innate immune response [21,23]. Leukocyte transmigration to the CNS is mainly stimulated by the chemokines produced by the epithelial cells of the CP. These cells serve as the BCSFB and determine the degree to which molecules and leukocytes can translocate from the blood to the CSF and CNS, participating in the upregulation of chemokines following a peripheral stimulus [23]. In fact, we observed a significantly increased gene expression of the chemokine CCL-5 and tendency to increase the gene expression of CXCL-10 in the CP of dogs with CanL. 

The chemokine CCL-5 is highly chemoattractant for macrophages and monocytes, as well as several subpopulations of lymphocytes, dendritic cells, and natural killer cells [24]. Moreover, CXCL-10 is induced by IFN-γ, which regulates the migration of Th1 effector cells to the site of inflammation during the adaptive immune response [24]. Previous studies that evaluated the profile of chemokines expressed in the CNS of dogs with CanL detected a pattern of upregulation in CCL-3, CCL-4, and CCL-5 [11]. These results agree with our findings, although there was no statistical difference in gene expression of CCL-3 and CCL-4 in the CP of infected dogs compared to the control group. Increased CCL-5 expression has also been found in the skin and spleen of dogs with leishmaniasis, and is positively correlated to parasite load [45,46]. Furthermore, it is important to consider the limitations of this study, such as the limited number of dogs and the use of dogs in different stages of infection, which may have impacted the lack of statistical significance in the results. In vitro studies should be ideal for verifying the observed results.

Since the presence of lymphocytes has already been reported in the brains of dogs with CanL [9], increased expression of CCL-5 and CXCL-10, as observed in our study, is probably related to lymphocyte recruitment for the CP. The presence of T lymphocytes in the CP could alter the function of the BCSFB or favor the production of other pro-inflammatory mediators that were not measured in our study. These mediators can promote the inflammatory phenotype of glial cells once they circulate in the CSF. 

## 5. Conclusions

Dogs with CanL frequently present with leptomeningitis and choroiditis regardless of the clinical stage of the animal. We confirmed blood–CSF barrier and BBB dysfunction due to the presence of anti-Leishmania antibodies and albumin in the CSF. We also observed the presence of CD3+ T cells migrating through the CP. In addition, the upregulation of the chemokine CCL-5 and the tendency to increase CXCL-10 may favor local inflammation during CanL infection. These findings, associated with the previously described dysfunction of the blood–brain and blood–cerebrospinal barriers in dogs with CanL (8), suggest that the CP may act as a gateway for parasites’ entrance into the CNS.

## Figures and Tables

**Table 1:** Percentage of dogs with inflammation classified according to intensity and region.

Intensity of Inflammation	Brain Region		
	Leptomeninges (%)	Subependyma (%)	Choroid Plexus (%)
0 (−)	0.0	42.1	5.3
1 (+)	21.1	36.8	15.8
2 (++)	36.8	21.1	47.4
3 (+++)	42.1	0.0	31.6

0—(−) Absence of inflammatory cells; 1—(+) Discreet or focal accumulation of rare cells; 2—(++) Diffuse or multifocal accumulation of few cells; 3—(+++) Marked presence of inflammatory cells.

## Data Availability

The original contributions presented in the study are included in the article/Supplementary Material. Further inquiries can be directed to the corresponding author/s. The datasets for this study can be found in the Unesp Institutional Repository [https://repositorio.unesp.br/handle/11449/151333 (accessed on 20 June of 2023)].

## Supplementary Materials

The following supporting information can be downloaded at: https://www.mdpi.com/article/10.3390/ani13132060/s1, Table S1: Serological, hematological and biochemical data from the dogs used in the study, clinical signs and clinical staging. Table S2: Histopathological evaluation according to region and intensity of inflammation.
