# Supplementary material for: Increased CCL-5 (RANTES) Gene Expression in the Choroid Plexus of Dogs with Canine Leishmaniosis
Source: Animals (Basel). 2023 Jun 22;13(13):2060. doi: 10.3390/ani13132060 (PMC10340004; doi:10.3390/ani13132060)
Supplement: Supplementary file 1 [file animals-13-02060-s001.zip › animals-2396932-supplementary.pdf]

animals

MDPI

Supplementary Materials

Supplementary Table S1. Serological. hematological and biochemical data from the dogs used in the study. clinical signs and clinical staging.

Dogs	Sex	ELISA O.D *Leishmania spp.* (cut-off > 0.270)	RBC x106/µl	Hb g/dl	HT (%)	WBC (/µl)	NEU (/µl)	LINF (/µl)	PPL x103/µl	PPT g/dl	ALT (U/L)	FA (U/L)	PT (U/L)	CREAT (mg/dl)	URE (mg/dl)	ALBUM (mg/dl)	Clinical Sings**	Clinical Staiging (Sollano-Gallego, 2009)
1	F	0.347	6.92	15.3	47	20200	17170	606	220	6.8	47	38	8.0	1.8	15	3.03	1	2
2	M	0.693	4.73	11.0	34	10700	7276	3210	220	9.0	42	293	8.8	0.9	28	1.54	1	1
3	F	0.904	5.2	11.3	35	14600	12848	1168	380	8.6	46	50	8.1	2.3	73	1.22	2	3
4	M	0.582	7.04	15.0	46	14500	10440	2900	320	7.7	15	19	5.4	0.9	21	1.82	2	2
5	M	0.329	2.26	4.3	14	40200	36180	402	240	6.6	12	50	6.0	2.4	85	0.89	3	4
6	F	0.656	4.24	9.0	28	16800	11928	3528	200	11.3	204	837	11.5	1.4	50	1.12	1	2
7	F	0.484	3.32	6.3	20	23900	21988	717	320	8.0	43	46	7.7	0.9	40	1.16	2	1
8	M	0.498	3.27	7.3	22	3800	3040	532	80	5.4	**	**	5.7	1.2	20	1.95	1	2
9	M	1.291	0.98	2.3	7	18600	74484	372	240	6.8	**	**	7.1	1.5	30	2.37	2	2
10	M	0.678	0.41	0.7	3	24500	23520	245	160	7.2	**	**	7.4	2.6	21	2.53	3	3
11	M	1.323	2.36	5.3	16	3400	2788	442	80	7.2	**	**	7.5	1.5	20	1.85	2	2

animals

MDPI

12	F	1.365	5.1	11.9	36	8700	**	900	70	8.0	**	**	6.5	0.6	14	1.37	1	1
13	M	0.754	4.3	10.2	31	11300	**	200	50	8.0	**	**	7.1	0.9	14	1.83	2	2
14	F	0.720	3.1	6.2	19	11900	**	3600	60	6.0	**	**	5.6	0.5	17	1.03	1	1
15	F	0.837	2.0	5.4	16	12600	**	100	20	5.4	**	**	5.6	0.4	7	0.70	1	1
16	F	0.533	4.2	10.8	32	9000	**	800	**	8.0	**	**	8.1	0.7	6	1.30	2	2
17	M	1.137	1.7	3.6	11	4900	**	1100	37	8.0	**	**	8.6	2.2	168	1.40	2	3
18	F	0.378	0.4	1.0	3	13500	**	6900	150	12.0	**	**	11.7	1.0	7	2.30	2	2
19	M	0.710	5.5	12.2	37	14900	**	300	110	9.0	**	**	9.5	1.0	8	1.60	2	2
Control 1	M	0.069	6.87	14.3	44	18500	13135	2590	300	7.0	40	28	5.8	0.9	24	2.66	***	***
Control 2	M	0.124	8.08	17.0	48	15300	9486	2295	220	7.3	72	21	6.7	1.0	26	4.12	***	***
Control 3	M	0.215	7.41	15.3	47	10600	5480	1200	220	8.7	42	27	7.7	1.4	23	2.71	***	***
Control 4	M	0.614	5.16	15.3	37	6600	5280	1000	320	8.6	15	19	8.4	1.2	108	2.00	***	***

• Values lower than references values;
• Values higher than references values.

**Clinical Signs:
1 – Lymphadenopathy and dermatological signs;
2 – Onychogryphosis. ulcers. weight loss;
3 – Cachexia. uveitis. arthritis. signs of chronic kidney disease (uremic breath. stomatitis).

animals

MDPI

Supplementary Table S2. Histopathological evaluation according to region and intensity of inflammation.

Dog	Intensity of Inflammation / Region			Perivascular Cuffs
	Leptomeninges	Subependyma	Choroid Plexus	
1	3	0	2	
2	3	2	3	+
3	1	0	1	
4	2	0	2	
5	3	1	3	+
6	3	1	2	
7	3	2	3	+
8	1	0	1	
9	2	0	2	
10	2	1	2	
11	1	0	1	
12	3	1	2	
13	2	1	2	
14	2	0	1	
15	3	2	3	+
16	2	1	2	
17	3	2	3	+
18	2	1	2	
19	1	0	0	

0 – (-) Absence of inflammatory cells;
1 – (+) Discreet or focal accumulation of rare cells;
2 – (++) Diffuse or multifocal accumulation of few cells;
3 – (+++) Marked presence of inflammatory cells.
(+) Presence of perivascular cuffs formed by at least two layers of inflammatory cells.
